# Strategies for Targeting SARS CoV-2: Small Molecule Inhibitors—The Current Status

**DOI:** 10.3389/fimmu.2020.552925

**Published:** 2020-09-18

**Authors:** Narasimha M. Beeraka, Surya P. Sadhu, SubbaRao V. Madhunapantula, Rajeswara Rao Pragada, Andrey A. Svistunov, Vladimir N. Nikolenko, Liudmila M. Mikhaleva, Gjumrakch Aliev

**Affiliations:** ^1^Department of Biochemistry, Center of Excellence in Molecular Biology and Regenerative Medicine (CEMR), JSS Academy of Higher Education & Research (JSS AHER), Mysore, India; ^2^AU College of Pharmaceutical Sciences, Andhra University, Visakhapatnam, India; ^3^Special Interest Group in Cancer Biology and Cancer Stem Cells (SIG-CBCSC), JSS Medical College, JSS Academy of Higher Education & Research (JSS AHER), Mysore, India; ^4^I. M. Sechenov First Moscow State Medical University of the Ministry of Health of the Russian Federation (Sechenov University), Moscow, Russia; ^5^Department of Normal and Topographic Anatomy, M.V. Lomonosov Moscow State University, Moscow, Russia; ^6^Research Institute of Human Morphology, Moscow, Russia; ^7^Sechenov First Moscow State Medical University (Sechenov University), Moscow, Russia; ^8^Institute of Physiologically Active Compounds, Russian Academy of Sciences, Moscow, Russia; ^9^GALLY International Research Institute, San Antonio, TX, United States

**Keywords:** SARS-CoV, SARS-CoV-2, COVID-19, natural Nrf-2 modulators, NSMIs

## Abstract

Severe Acute Respiratory Syndrome-Corona Virus-2 (SARS-CoV-2) induced Coronavirus Disease - 19 (COVID-19) cases have been increasing at an alarming rate (7.4 million positive cases as on June 11 2020), causing high mortality (4,17,956 deaths as on June 11 2020) and economic loss (a 3.2% shrink in global economy in 2020) across 212 countries globally. The clinical manifestations of this disease are pneumonia, lung injury, inflammation, and severe acute respiratory syndrome (SARS). Currently, there is no vaccine or effective pharmacological agents available for the prevention/treatment of SARS-CoV2 infections. Moreover, development of a suitable vaccine is a challenging task due to antibody-dependent enhancement (ADE) and Th-2 immunopathology, which aggravates infection with SARS-CoV-2. Furthermore, the emerging SARS-CoV-2 strain exhibits several distinct genomic and structural patterns compared to other coronavirus strains, making the development of a suitable vaccine even more difficult. Therefore, the identification of novel small molecule inhibitors (NSMIs) that can interfere with viral entry or viral propagation is of special interest and is vital in managing already infected cases. SARS-CoV-2 infection is mediated by the binding of viral Spike proteins (S-protein) to human cells through a 2-step process, which involves Angiotensin Converting Enzyme-2 (ACE2) and Transmembrane Serine Protease (TMPRSS)-2. Therefore, the development of novel inhibitors of ACE2/TMPRSS2 is likely to be beneficial in combating SARS-CoV-2 infections. However, the usage of ACE-2 inhibitors to block the SARS-CoV-2 viral entry requires additional studies as there are conflicting findings and severe health complications reported for these inhibitors in patients. Hence, the current interest is shifted toward the development of NSMIs, which includes natural antiviral phytochemicals and Nrf-2 activators to manage a SARS-CoV-2 infection. It is imperative to investigate the efficacy of existing antiviral phytochemicals and Nrf-2 activators to mitigate the SARS-CoV-2-mediated oxidative stress. Therefore, in this review, we have reviewed structural features of SARS-CoV-2 with special emphasis on key molecular targets and their known modulators that can be considered for the development of NSMIs.

## Introduction

### Global Burden of COVID-19

COVID-19 is a devastating disease caused by a coronavirus related to the one that caused outbreaks of Severe Acute Respiratory Syndrome (SARS) in the year 2002 (1, 2). Middle East Respiratory Syndrome (MERS)-related coronavirus is an infamous member of this cohort. COVID-19, which is caused by the SARS-CoV-2 infection, was detected in Wuhan, China in December 2019. The World Health Organization (WHO) declared this infection a pandemic on March 11 2020 due to its severity and rapid spread across the globe. As of June 11 2020, SARS-CoV-2 had infected 7.4 million individuals, and caused 4,17,956 deaths across 212 countries worldwide (Table 1).

**Table 1:** Recent statistics of SARS-CoV2 infection—Top 10 countries.

**Country**	**Infected (in Millions) (%)^#^**	**Recovered (in Millions) (%)^$^**	Deaths (in Thousands) (%)^*^
United States of America	2.064 (0.623)	0.800 (38.79)	115,115 (5.57)
Brazil	0.772 (0.363)	0.380 (49.23)	39,680 (5.13)
Russia	0.493 (0.338)	0.252 (51.20)	6,358 (1.28)
United Kingdom	0.290 (0.427)	0.135 (46.52)	41,128 (14.17)
Spain	0.289 (0.618)	Not available	27,136 (9.37)
India	0.287 (0.020)	0.140 (49.09)	8,107 (2.82)
Italy	0.235 (0.389)	0.169 (72.08)	34,114 (14.46)
Peru	0.208 (0.633)	0.098 (46.94)	5,903 (2.82)
Germany	0.186 (0.22)	0.170 (91.34)	8,844 (4.73)
Iran	0.177 (0.212)	0.140 (79.01)	8,506 (4.78)

# *Percentage of total population*.

$ *Percentage of total infected cases*.

* *Percentage of total infected cases*.

*List of top 10 countries in the world affected with COVID-19*.

### Structural Features of SARS-CoV-2

**Figure 1:**
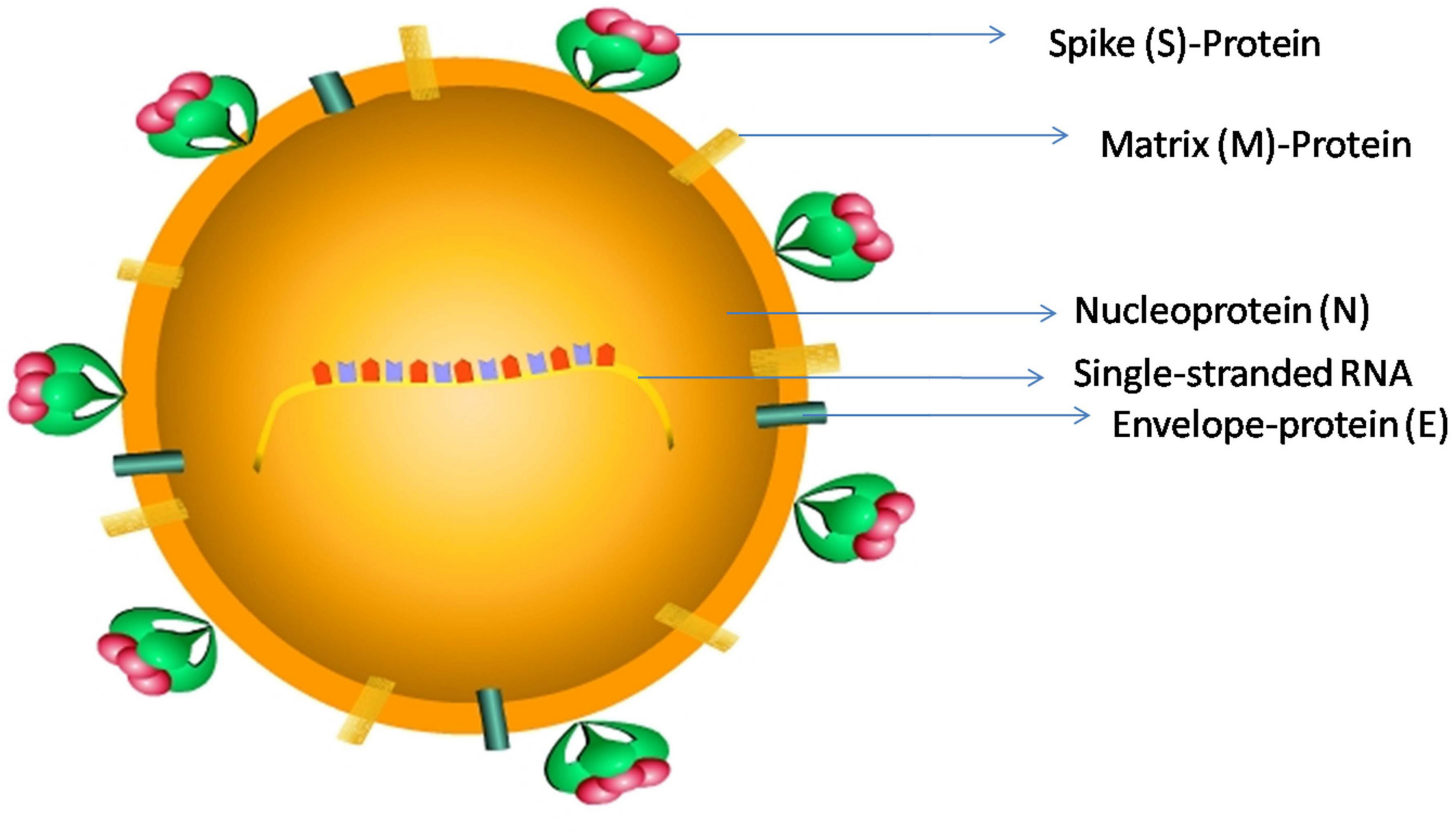
The schematic representation of SARS-CoV-2 structure: SARS-CoV-2 has a size ranging from 60 to 140 nm, and is a spherical to elliptical shaped virus with a crown-like appearance; it consists of a single-stranded RNA genome, a Spike protein (S), a Matrix protein (M), a nucleoprotein (N), and an Envelope-protein (E).

Coronaviruses (CoV) belongs to a family of single-stranded RNA viruses (+RNA) that can infect a variety of mammals such as bats and humans (3). SARS-CoV-2 contains RNA of 29,891-nucleotide length, which codes for 9,860 amino acids (4). The RNA has a 5' cap and 3' poly-A tail and produces a poly-protein 1a/1ab (pp1a/pp1ab) in the host (4). SARS-CoV-2 belongs to beta CoV category and appears in a crown shape with a size of ~60–140 nm (Figure 1).

Gene sequencing data revealed that SARS-CoV-2 has 89 and 82% sequence similarity with bat SARS-like-CoV-ZXC21 and human SARS-CoV, respectively (4, 5). The spike (S) protein-coding gene mutation in the *nsp2 and nsp3* regions results in the replacement of glycine (G) with serine (S) at 723 position (G723S), and an isoleucine (I) replaced with proline (P) at 1010 amino acid position (I1010P). Due to these mutations, the invading potential of SARS-CoV-2 has increased significantly toward host tissues. This virus can also be transmitted through the respiratory droplets from coughs and sneezes of infected individuals (4). This mode of aerosol transmission is possible, especially, when protracted exposure occurs in closed areas (4). The incubation time of the virus varies significantly from individual to individual. In general it takes about 6 days from the day of infection to the first appearance of symptoms. However, in a few cases the symptoms may appear only after 2 weeks (6).

### SARS-CoV2 Infection and Pulmonary Pathogenesis

Members of *Coronaviridae* are known to induce respiratory complications in humans (7, 8). At first, SARS-CoV, MERS-CoV, and SARS-CoV-2 varieties were transmitted from animals to humans which triggered severe respiratory diseases (9–11). However, subsequent transmission occurred among humans primarily due to physical contact. Hence, conventional preventive measures such as physical isolation were implemented to avoid propagation of early infection across the human population (1, 12). Similar to the SARS-CoV, the pathological manifestations of SARS-CoV-2 could induce lung malfunction in humans as indicated by the severe acute respiratory syndrome and pneumonia (12). Recent studies reported that SARS-CoV-2 infection can induce mild, moderate, and severe illness in infected patients (4). Clinical manifestations of this infection include chronic pneumonia, sepsis, septic shock, fever, and dry cough (4). A progressive respiratory failure during this infection may lead to sudden death (4). Mild illness resulting from a SARS-CoV-2 infection is characterized by the presence of malaise, headache, low fever and dyspnea. In the case of moderate illness from SARS-CoV-2, the complication is manifested by the presence of cough and mild pneumonia. Severe illness from SARS-CoV-2 is associated with chronic pneumonia, cough, SARS, hypoxia, and tachypnea (in children) followed by respiratory, and cardiovascular system failure (4). The autopsy and biopsy reports of SARS-CoV-2 patients revealed severe edema with pulmonary tissue exudates, focal reactive hyperplasia, damage to pneumocytes as well as alveolar macrophages, and patchy cellular infiltration (13).

Coronavirus-induced lung damage has been demonstrated experimentally by several investigators in animal models (14). For instance, the Sialodacryoadenitis virus and Parker's RCoV were shown to induce damage to alveolar type-I cells through the expression of pro-inflammatory cytokines, and chemokines such as *CINC-2, CINC-3, LIX, MIP-3*α*, and fractalkines* (15–21). For example, fractalkine promotes the infiltration of cytotoxic lymphocytes in the alveolar epithelium thereby inducing a severe inflammatory response (15, 22). Similarly, MIP-3α confers the chemotaxis of immune cells via IL-1β and TNF-α inflammatory mediators (17, 22–25). Therefore, these animal models could be used to develop effective pharmacological agents against SARS-CoV-2 infections.

### Molecular Mechanisms of SARS-CoV-2 Infection

Studies from several laboratories have demonstrated that the entry of SARS-CoV-2 into human cells is facilitated by ACE-2 (26). ACE-2 is a member of the Renin-angiotensin system (RAS), which plays a vital role in cardiovascular and renal homeostasis. ACE-2 and *TMPRSS2* facilitates the entry of the virus into host cells during SARS-CoV-2 infection (7). In addition, there are other proteases such as aminopeptidase N (APN) which plays a prominent role for the entry of HCoV-NL63 and HCoV-229E into host cells (27–30). APN is a membrane-bound glycoprotein that mediates the zinc-dependent protease activity during the entry and or replication of coronavirus strains into host cells (29, 31, 32). Hence, the ACE-2 receptor's down-modulation may prevent SARS-CoV-2 viral entry/replication (33). The S-protein of SARS-CoV and other coronavirus strains are different in their structural and functional domains (3). S-protein can bind to the N-terminus of ACE-2 receptors on the outer surface of host cells including respiratory epithelium of the lungs (34–36). Identifying the key amino acid residues in S-protein of the SARS-CoV-2 strain may benefit virologists and medical scientists to develop better therapeutic agents. However, to date these details are not known, hence, there is an immediate requirement to identify the amino acids involved in binding S-proteins to ACE-2 receptors on host cell surfaces. Furthermore, investigations should also focus on establishing the structural similarities of S-protein motifs that are interacting with the ACE-2 receptors of other coronavirus strains (37–41). These investigations might help in deciphering molecular strategies to target receptor binding sites of ACE-2 proteins with SARS-CoV-2 using novel therapeutics and vaccines to avoid membrane fusion process and viral entry (7).

The TMPRSS2 protease can foster the entry of the SARS-CoV-2 virus by activating the S-protein for virus-host cell membrane fusion, consequently enhancing viral replication in the host cells (7, 42–46). TMPRSS2 plays a vital role in generating inflammatory cytokines and chemokines in lung epithelial cells by cleaving S-protein during coronavirus infections including SARS-CoV-2. Hence, TMPRSS2 is another potential therapeutic target to consider for the novel drug development against SARS-CoV-2 (46–48).

### Novel Small Molecule Inhibitors (NSMIs) in the Prevention and Treatment of SARS-CoV-2 Infections

Prevention and treatment of SARS-CoV-2 infections are achieved at different levels (49). The primary approach involves physical isolation to prevent the spread of virus from individual to individual; the second approach involves inhibiting the entry of virus into human cells and the third method includes treating the infected individuals to minimize inflammatory reactions and pulmonary damage. Although physical isolation is the ideal way of limiting the spread, in reality this approach is difficult to execute, hence, many pharmacological companies are actively involved in developing small molecule inhibitors to prevent the entry of the virus into human hosts (7, 49). In this regard several NSMIs have been investigated to treat SARS-CoV; but, significant breakthroughs are yet to come for treating SARS-CoV-2 (48) (Table 2).

**Table 2:** Structure and probable mechanism of action of NSMIs against SARS-CoV-2.

**Small Molecule Inhibitors *Predicted* to be effective against SARS-CoV-2**	**Mechanism of Action**	**Structure**	**References**
SiRNA	Targets Orf7a required for viral assembly (or) Targets Orf7b (or) Targets Orf3a required for viral budding and release Note: SiRNA is yet to be examined against SARS-CoV-2 infection	–	(50)
GRL0617	Targets non-structural proteins nsp3 (Papain like proteinase) Note: Yet to be examined against SARS-CoV-2 infection	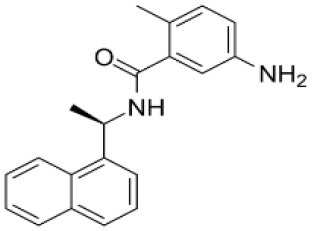	(51)
Benzodioxolane derivatives	Targets non-structural proteins nsp3 (Papain like proteinase) in coronavirus Note: Yet to be examined against SARS-CoV-2 infection	1-[(R)-1-(1-Naphthyl)ethyl]-4-[3,4- (methylenedioxy) benzylamino] carbonylpiperidine1-[(S)-1-(1-naphthyl) ethyl]-4-[3,4-(methylenedioxy)benzylamino] carbonylpiperidine	(52)
5-chloropyridinyl indolecarboxylate	Targets non-structural proteins nsp5 (3C-like main protease in SARS coronavirus) required for replicase synthesis Note: Yet to be examined against SARS-CoV-2 infection	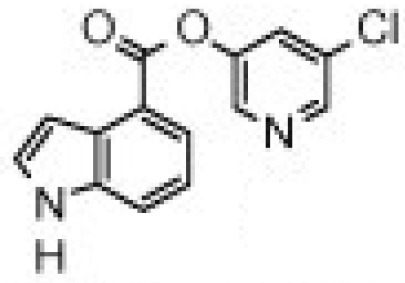	(53)
2978/10 humanized antibodies	Mitigate SARS-CoV infection by targeting virus-neutralizing epitopes	–	(54)
Amiodarone	Targets SARS-CoV by inhibiting endosomal processing in host cells Note: Clinical Trials are at Recruiting Stage to test against SARS-CoV-2 infection—NCT04351763	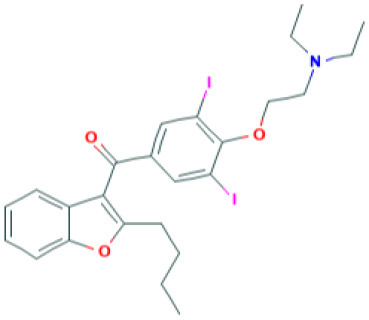 *(Pubchem)*	(55)
Arbidol	Targets S-protein of SARS-CoV and prevent viral fusion Note: Clinical Trials are at Recruiting Stage to test against SARS-CoV-2 infection -NCT04255017	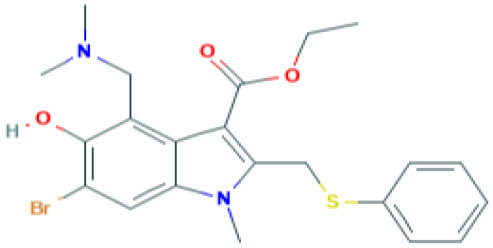 *(Pubchem)*	(56)
TSL-1	Targets SARS-CoV replication Note: Yet to be examined against SARS-CoV-2 infection	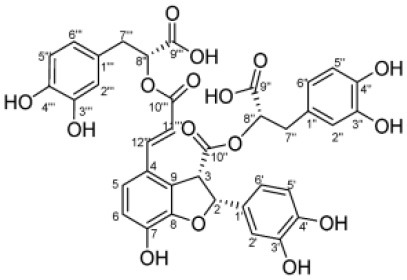	(57) (58)
TACE inhibitor (TAPI-2)	Blocks SARS-CoV replication in lungs Blocks ACE2 shedding Note: Yet to be examined against SARS-CoV-2 infection	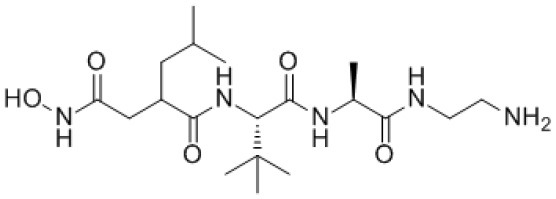	(59)
IFN—α B/D	Blocks SARS-CoV replication in lungs		(60)
IFN-β and-γ	Blocks SARS-CoV replication in lungs Note: Completed Clinical Trials for Interferon Beta-1A and Interferon Beta-1B -NCT04343768 Clinical Trials are at Recruiting Stage to test against SARS-CoV-2 infection-NCT04324463; NCT04350281 (IFN-β)		(61) (62) (63)
Camostat	TMPRSS2 serine protease Inhibitor in SARS-CoV-2 infection Note: Clinical Trials are at Recruiting stage to test against SARS-CoV-2 infection—NCT04321096	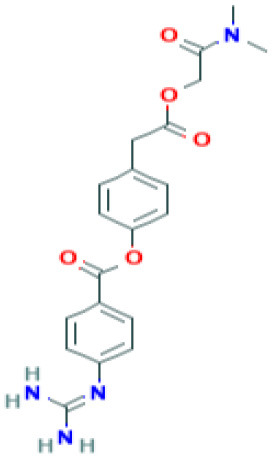 *(Pubchem)*	(7)
Nafamostat	TMPRSS2 serine protease Inhibitor in SARS-CoV-2 virus Note: Yet to be examined against SARS-CoV-2 infection in clinical trials	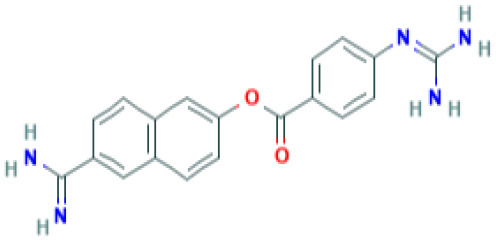 *(Pubchem)*	https://www.eurekalert.org/pub_releases/2020-03/tiom-nie032420.php
Pegylated IFN-α	Blocks SARS-CoV replication in lungs Note: Yet to be examined against SARS-CoV-2 infection	–	(2)
Remdesivir	Effective against SARS-CoV-2 infection *in vitro* Note: Clinical Trials—Recruiting stage to test against SARS-CoV-2 infection - NCT04365725	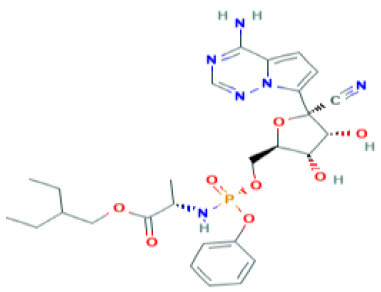 *(Pubchem)*	(64)
Lopinavir	Predicted to block SARS-CoV-2 M^pro^ (Molecular docking studies) Note: Clinical Trials are at Recruiting stage to test against SARS-CoV-2 infection-NCT04364022	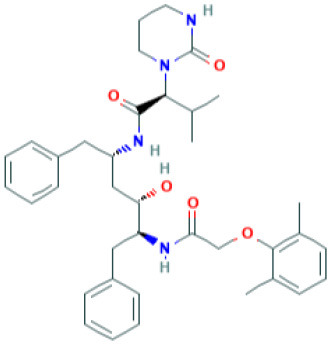 *(Pubchem)*	(65)
Nelfinavir	Predicted to block SARS-CoV-2 M^pro^ (Molecular docking studies)	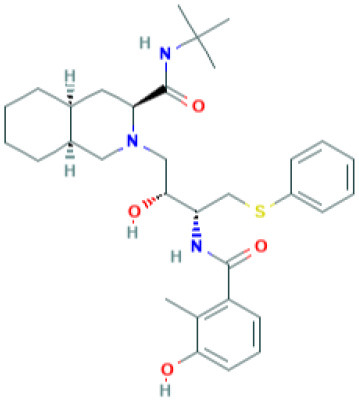 *(Pubchem)*	(66)
Tocilizumab	Block SARS-CoV-2 viral induced cytokine storm—IL-6 receptor-targeted monoclonal antibody (mAb) (Ongoing clinical trials in China and Italy)—ChiCTR2000029765; NCT04377750; NCT04377659	–	**doi.org/10.1038/s41577-020-0308-3**
SSAA09E1[[(Z)-1-thiophen-2-ylethylideneamino]thiourea]	Blocks cathepsin L required for SARS-CoV processing Note: Yet to be examined against SARS-CoV-2 infection	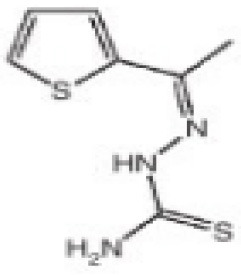	(67)
SSAA09E2N-[[4-(4-methylpiperazin-1-yl)phenyl]methyl]-1,2-oxazole-5-carboxamide	Blocks SARS-CoV interaction with ACE-2 Note: Yet to be examined against SARS-CoV-2 infection	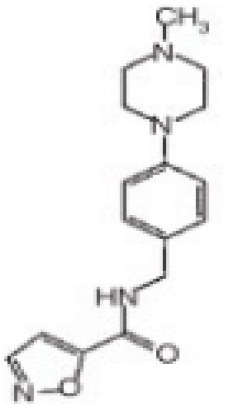	(67)
SSAA09E3[N-(9,10-dioxo-9,10-dihydroanthracen-2-yl)benzamide]	Blocks SARS-CoV fusion to host cell membrane Note: Yet to be examined against SARS-CoV-2 infection	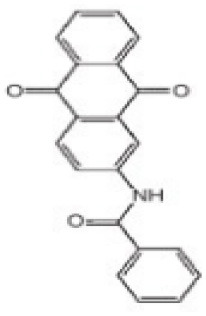	(67)

*NCT numbers were obtained from https://clinicaltrials.gov/*.

#### Viral Entry Inhibitors vs. SARS-CoV-2

**Figure 2:**
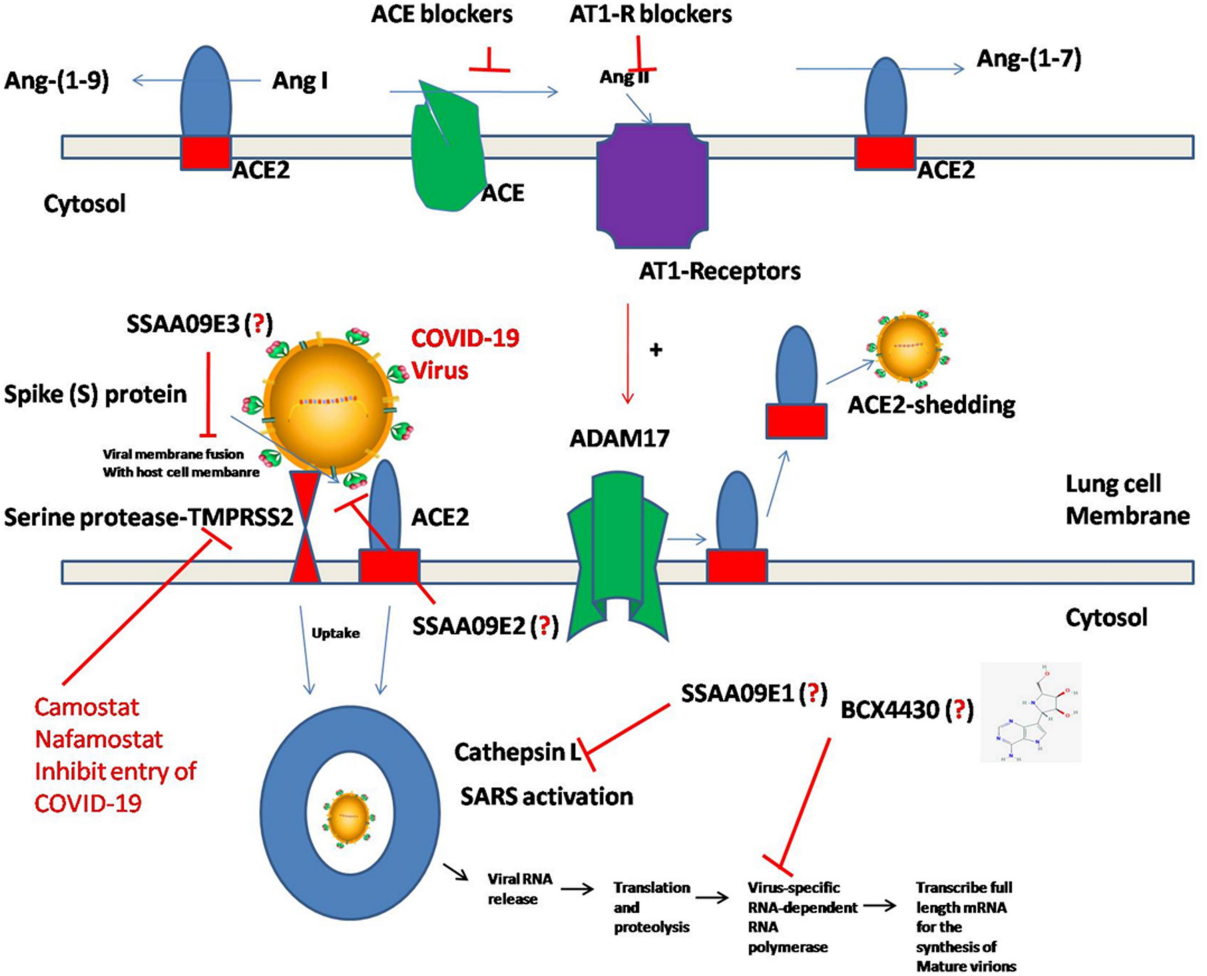
Molecular pathogenesis of SARS-CoV-2 in human lung cells. Binding of S-protein of SARS-CoV-2 to the ACE-2 receptors triggers the processing of ACE-2 through ADAM-17/TNF-α-converting enzyme and induces the “ACE-2 shedding” into the extracellular space and facilitates uptake of SARS-CoV-2 followed by the development of SARS. Alternatively, the entry of SARS-CoV-2 by membrane TMPRSS2 serine protease'/HAT (Human Airway Trypsin-like protease)-mediated cleavage of ACE2 can facilitate SARS-CoV S-glycoprotein-mediated virus entry. Even though, several NSMIs targeting these processes were described and their mode of action against coronavirus were delineated, their efficacy against SARS-CoV-2 is yet to be tested.

Adedeji et al. (49) reported the discovery and characterization of novel inhibitors to block SARS-CoV replication via different mechanisms. One mechanism uses screening of small molecule inhibitors using “*HIV-1 pseudotyped with SARS-CoV surface glycoprotein S (SARS-S)*” (49, 68). “SSAA09E2” is a novel small molecule inhibitor, which blocks the interaction of CoV SARS-S with ACE-2 receptors, thus blocking the viral entry (49). Another NSMI is “SSAA09E1” reported to be involved in blocking the cathepsin L, which is required for CoV-SARS-S processing to mediate viral entry into the host cell (49). SSAA09E3 is another NSMI, which can block the fusion of viral membranes with host cell surfaces (49) (Figure 2). Since the pathological aspects and genomic similarity of SARS-CoV-2 virus with SARS-CoV, the above strategies of inhibition may be considered for developing potent pharmacological agents to prevent SARS-CoV-2 infections (49). However, the prospective research should address the efficacy of these inhibitors against SARS-CoV-2 infections.

#### Kinase Inhibitors vs. SARS-CoV-2

Cytokine storm was predominantly reported during SARS-CoV-2 infection. Targeting cytokine-mediated inflammatory responses induced by SARS-CoV-2 is another viable approach for mitigating the complications of viral infection. In this regard, Chang et al. (35), documented the inflammatory cascades mediated through intracellular signaling pathways conferred by the SARS-CoV in both lung epithelial cells and fibroblasts. Authors of this study have reported that S-protein of SARS-CoV efficiently mediate the IL-8 release in the infected lung cells by activating MAPKinases, and activator protein-1 (AP-1) without intervention of NF-kB cascade (35). This study suggested a promising lead for novel rational drug design through the identification of a “specific sequence motif of S-protein functional domain,” which is responsible for inducing IL-8-mediated inflammatory response in lungs (35). Baricitinib is a pharmacological agent, which was reported to block the SARS-CoV-2 viral entry and inflammation through the inhibition of AP2-associated protein kinase 1 (AAK1), cyclin g-associated kinase, and janus kinase-1 and 2 (69). Chloroquine (CQ) and hydroxychloroquine (HCQ) were reported to be effective in mitigating the coronaviral load (70, 71). CQ and HCQ not only inhibit the entry of SARS-CoV-2 but also change the pH of acidic intracellular organelles such as endosomes and lysosomes thereby preventing membrane fusion reactions. However, many contradictions and queries prevail pertaining to the use of HCQ for the treatment of COVID-19. At the time of the submission of this review, results of many clinical trials are yet to be announced, hence, the efficacy of HCQ for inhibiting SARS-CoV-2 infection is still a possibility.

Prospective studies should focus on testing the FDA approved inhibitors of “ABL-1 kinases,” “PI3K/Akt/mTOR” signaling, and “MAPKinase” pathways against SARS CoV-2. Since these pathways are involved in cell survival, inflammatory cytokines production, and proliferation of cells, targeted downregulation of these pathways is likely to mitigate the exacerbations induced by coronavirus. In this direction, many of these inhibitors are currently being tested against SARS-CoV-2 (Table 3) (72–74). For instance, sorafenib, which inhibits RAF, is being experimented in preclinical models and early clinical trials (72). Likewise, the efficacy of IL-1 receptor antagonists and TNF-α receptor antagonists for blocking the rat coronavirus-mediated chemokine production was already proven effective in animal models (15, 75, 76). Further studies testing the safety and efficacy are warranted before considering these inhibitory agents for treating individuals infected with SARS-CoV-2 (76).

**Table 3:** Ongoing clinical trials against SARS-CoV2 using MAPKinase Inhibitors.

**MAPK Inhibitor**	**Mechanism of Action**	**References**	**Current Status of Clinical Trials against SARS CoV-2**
Trametinib Selumetinib	Inhibits MAPK/ERK—kinase family proteins viz. MEK1/2 inhibitor—Inhibits MAPK/ERK—kinase family Investigational (Phase III) MEK1/ERK1/2 inhibitor	(72) (72)	Ongoing Ongoing
Everolimus Miltefosine Teriflunamide Leflunomide	Inhibits PI3K/Akt/mTOR Kinases—Akt/mTOR Inhibits viral block of cell stress response and apoptosis	(73)	Ongoing
Dasatinib Imatinib Nilotinib	Inhibition of actin motility Blocks ABL1 kinase.	(73) (74)	Ongoing
PD98059 SB308520 SP600125	MEK inhibitor p38 inhibitor JNK inhibitor ^*^MOA: Inhibits SARS-CoV S protein-induced IL-8 Promoter activity using above MAPK cascade inhibitors	(35)	–
Chloroquine (NCT04351724) Hydroxychlorquine (NCT04352933)	Inhibits p38 MAPK activation and blocks viral replication	(70, 71)	Ongoing

* *Mechanism of action*.

However, the concept of “One Drug to Treat All” should be followed to combat several devastating viral infections (77, 78). For instance, the Ebola, Marburg, and SARS-CoV-2 are undoubtedly devastating viral pathogens, which can induce high mortality as they transmit rapidly via air and body fluids (78). Outbreaks of these viruses occur sporadically and currently there are no clinically approved NSMIs available to combat these viruses. A recent report by Taylor et al. (79) demonstrated the efficacy of a synthetic adenosine analog, BCX4430 in blocking a broad spectrum of viral species viz., “coronaviruses, paramyxoviruses, and bunyaviruses” as these viruses could induce SARS, measles, and mumps. BCX4430 could efficiently block both Ebola and Marburg viral titers in non-human primate models by targeting viral RNA polymerase (78, 79). Hence, this molecule should be tested for further studies against SARS-CoV2 infections in humans.

#### Membrane Protease Inhibitors vs. SARS-CoV-2

Targeting the membrane protease involved in viral S-protein processing and the viral entry into host cells is another approach in mitigating SARS-CoV-2. The host cellular proteases viz., “*trypsin*,”, “*miniplasmin*,” “*human airway trypsin like protease*,” “*tryptase Clara*,” and “*TMPRSS2*” could cleave the HA glycoprotein located in influenza A virus and thereby promote viral entry into lung cells (80). The usage of serine protease inhibitors such as Camostat and Aprotinin significantly blocked the replication of influenza virus in epithelial cells of lungs and bronchioles (81). In addition, these NSMIs could block the release of inflammatory mediators such as cytokines, IL-6 and TNF-α, during this infection (81).

TMPRSS2 is a key protein involved in the pathogenesis of several seasonal viral infections including influenza, H1N1, H3N2, and H7N9 (82–85). TMPRSS2 cleaves the S-protein of coronavirus to produce unlocked, fusion-catalyzing viral forms and binds to the host cell surface thereby enhancing rapid viral entry (43, 44, 86–90). Both SARS-CoV and MERS-CoV could rapidly enter into the host cells as TMPRSS2 can facilitate viral binding to the cell surface (42, 43, 45, 87, 91, 92). TMPRSS2 also plays a vital role in the immuno-pathology of coronavirus infections including SARS-CoV-2 across lungs by inducing lung fibrosis (46). Hence, the emerging research should promote the development of NSMIs to target these proteases thereby hindering the entry of SARS-CoV-2 into host cells.

A proof-of-concept study by Iwata-Yoshikawa et al. (46) reported that SARS-CoV failed to replicate in the bronchioles and lungs of TMPRSS2 knockout mice. Authors of this study reported elevated expression of TLR3-mRNA expression in the lungs of “SARS-CoV-inoculated TMPRSS2-deficient mice” and showed enhanced TLR-3 mediated localization of dsRNA into endosomes (46). In this study, TMPRSS2 knockout has resulted in downregulation of inflammatory cytokines and chemokine expression, which are involved in the bronchiolitis obliterans organizing pneumonia (BOOP), SARS, and pulmonary fibrosis in SARS-CoV infection (46, 93, 94).

#### Interferon Therapy vs. SARS-CoV-2

The intricate SARS-CoV-2 pathogenesis is similar to that of SARS-CoV. Studies have reported the efficacy of IFNs to block SARS-CoV in cell line models but not against SARS-CoV-2. Among IFN- α/ -β/ and -γ, the IFN-β was reported to be the most potent blocker of SARS-CoV growth (3, 95–98). Furthermore, IFN-β and-γ have a synergistic effect in blocking SARS-CoV viral replication (62, 63). However, the effect of this combination against SARS-CoV-2 is not yet reported. Therefore, future studies should focus on determining the efficacy of IFN- α/ -β/ and –γ against SARS-CoV-2 infections.

#### SiRNAs vs. SARS-CoV-2

Unlike small molecule inhibitors, siRNAs are specific and can be designed to mitigate SARS-CoV *associated structural proteins* by targeting ORF4 (99, 100), ORF5 (101, 102), ORF9a (50, 103, 104), and ORF7a (50, 105–107). For example, siRNAs siSC2 and siSC5 have shown success in cultured cells as well as in preclinical mouse models in inhibiting the SARS infection without causing toxicity (108). Several other reports have also recently demonstrated the efficacy of SiRNAs to inhibit the expression of SARS-CoV genes coding for 3CL protease in cell line models (108–114). The activity of SARS-CoV 3CL protease is essential for viral replication as this protein is involved in the processing of viral proteins (114). Selective optimization and screening of hexa-chlorophene analogs can be “*active 3CL protease inhibitors*” during a SARS-CoV infection (114). Hence, the pharmacological agents/SiRNAs targeting these pathways may likely produce effective clinical outcomes in SARS-CoV-2 infections. However, clinical studies should test the utility of these agents/siRNA in reducing the burden of infections caused by SARS-CoV-2 (111).

#### Monoclonal Antibodies (MABs) and Other NSMIs vs. SARS-CoV-2

The genome of coronaviruses is reported to be significantly involved in coding both structural proteins, and non-structural proteins (nsp's) for the effective viral replication (115). The nsp's (nsp8C and nsp7) are required for novice CoV viral particle formation through viral ORF 1ab polyprotein processing (115). Several NSMIs were reported to target these non-structural proteins in coronavirus infections to treat SARS (115). For instance, GRL0617, a bendioxolane derivative, could target papain-like proteinases like nsp3 (51, 52, 116, 117), whereas 5-choloropyridinyl indolecarboxylate targets nsp5 (53, 118–120) and a “combination of zinc derivatives with pyrithione” targets nsp12 (121, 122); ranitidine bismuth citrate targets nsp13 (123–127). Monoclonal antibodies; CR3014 (128), mAb-201 (129), mDEF-201 (130), ampligen (131), polyICLC (61, 132), stinging nettle lectin (131), and TAPI-2 (a TACE-inhibitor) (59) are anti-coronaviral agents tested *in vivo* models of SARS. For instance, a study showed that Amiodarone (a known anti-arrhythmic agent) effectively targets coronaviral spreading in *in vitro* models (55). Working in a similar fashion, 2878/10 humanized antibodies can neutralize coronaviruses thereby reduce the complications caused by viral infections (54). However, the above NSMIs should be tested against SARS-CoV-2 viral associated proteins and against the activity of nsp's to derive an effective therapeutic intervention. Prospective research must focus on the development of novel “*helicase inhibitors, viral attachment inhibitors, and activity of Rhesus* θ*-defensin*” that block SARS-CoV-2 infection using *in vitro, in vivo*, and clinical studies (115). Hence, the development of NSMIs to target the synthesis of nsp's in SARS-CoV-2 may deliver cellular antiviral responses by blocking their replication in host cells (115, 133, 134).

#### Drug Repurposing Strategies (DRS) vs. SARS-CoV-2

Repurposing existing drugs is another strategy widely under consideration to target key proteins involved in the SARS-CoV-2 infection. In this regard, the existing NSMIs viz., antivirals (*umefenovir, remdesivir, Nitazoxanide, favipiravir, ritonavir, lopinavir, IFNs*), anticytokines, antimalaria drugs (*chloroquine, hydroxychloroquine*), and passive antibody therapies are currently being evaluated to improve clinical outcomes in SARS-CoV-2 infected patients (3, 47, 64, 135, 136). However, these agents require additional experimental and clinical validations before being tested in SARS-CoV-2 infections. For example, hydroxychloroquine (anti-malarial drug) and the tocilizumab (immunosuppressive drug) are preferred currently to mitigate viral entry and cytokine production in the SARS-CoV-2 infection. These drugs are being tested in ongoing trails in China and Italy (135, 137).

Priming the Spike (S)-protein of coronavirus by host cells using membrane proteases is a necessary process for viral entry and replication, which further determines zoonotic potential of coronaviruses (138). A recent report by Markus Hoffmann et al. (7) investigated the protease dependence of SARS-CoV-2 for its entry into cells. For example, SARS-CoV-2 uses the TMPRSS2 protease for its priming (7). Inhibition of TMPRSS2 using Camostat mesylate retarded the viral entry into Caco-2 cells (7). Camostat mesylate could be recommended as an NSMI for human clinical trials to combat the SARS-CoV-2 virus (7). This report delineated the ability of neutralizing antibody responses against S-protein to block the SARS-CoV-2 entry into host cells (139). The serum antibody responses raised to combat the “SARS-S protein/ACE-2 interface” during the SARS-CoV-2 infection indicates that the vaccination strategy may be an effective therapeutic modality against the COVID-19 infection (7).

### Conflicting Reports About the ACE-2 Inhibitors Usage for Treating SARS-CoV-2 Infections

ACE-2 catalytic efficacy is significantly higher than ACE for Angiotensin-II (140). Several compounds, such as MLN-4760, were screened according to structure-based/substrate-based studies through virtual screening for inhibiting ACE-2 activity (140–143). ACE-2 is predominantly expressed in lungs, brain, heart, blood vessels, and renal organs (144, 145). ACE-2 is essential for cardiovascular homeostasis, and CNS homeostasis as ACE-2 confer redox homeostasis by mitigating Ang-II-induced oxidative stress (146). However, in COVID-19, ACE-2 acts as receptor on human respiratory epithelial cells for SARS-CoV-2 binding (7). A recent report by Markus Hoffmann et al. (7) provided evidence that the SARS-CoV-2 strain use its spike (S)-protein to bind to ACE-2. Authors of this paper have also demonstrated the efficiency of TMPRSS2 in SARS-CoV-2 viral strain priming in host cells (7). Therefore, targeting ACE-2 could be a viable strategy to prevent the entry of SARS-CoV-2 into the human system. However, a recent report by Guan et al. (147) cautioned that the administration of ACE inhibitors significantly induced adverse clinical outcomes in COVID-19 patients due to severe hypertension, coronary artery disease, and chronic renal failure; hence, further use of ACE inhibitors to treat COVID-19 infections was halted (147–149). In another report Diaz (149) hypothesized that COVID-19 patients receiving I.V. infusions of ACEIs and ARBs (AT1- Receptor Blockers) are at a higher risk of attaining severe disease pathogenesis. Hence, they supported the development of NSMIs such as “TMPRSS2 inhibitors to treat SARS-CoV-2 infections (7)”.

### Reasons for the Failure of Current Therapeutic Modalities Against SARS-CoV-2

The failure of disease management and lack of selective therapies could be due to the intricate COVID-19 pathogenesis induced by the SARS-CoV-2 infection. Hence, the early recognition of disease is essential for effective management of COVID-19 (48).

Although, several reports delineated the efficacy of certain NSMIs viz., *ribavirin, promazine*, and *IMP dehydrogenase inhibitors* to inhibit *in vivo* models of SARS- CoV replication, later, they were proven ineffective (60, 150–152). A report by Reghunathan et al. (153) showed that the immune response produced against SARS-CoV may be different from other viral infections as indicated by the lack of upregulation in MHC-I genes, cytokines, and IFNs or complement-mediated cytolysis in peripheral blood mononuclear cells (PBMCs). The failure in the development of a vaccine is due to antibody-dependent enhancement and Th-2 immunopathology (154–156).

Pegylated IFN-α inhibits viral replication of SARS-CoV and offers protection against type I pneumocytes in lungs (2). A significant reason for the failure or lack of selective therapies against SARS-CoV-2-induced SARS is the intricate immune system mediated pathophysiology (4). Other reports by Law et al., also detailed similar mechanisms (157, 158). SARS-CoV can evade host IFN-mediated viral growth inhibition by activating IFN-regulatory factor 3 (157). Furthermore, SARS-CoV could induce apoptosis in lymphocytes *in vitro* using “*ORF 7a, ORF 3a, and ORF 3b, E protein, and N protein*” (159–162). For instance, the SARS-CoV can evade immunity as indicated by the decline in CD4 and CD8 T cells (163). Therefore, it is necessary to uncover the complement-based cytolysis in human patients in response to the SARS-CoV-2 strain as this virus executes unusual mechanisms to evade the human immune system consequently inducing pathogenesis and mortality. The prospective research should focus on this viral-mediated immune signaling with respect to SARS-CoV for developing effective NSMIs.

Intravenous (IV) hyperimmune globulin therapy is one of the immunotherapies known to downmodulate pro-inflammatory cytokines and mitigate the severity of infection in COVID-19 patients. IV infusion of immunoglobulins composed of a high dose of antibodies, which can bind to a number of inhibitory receptors viz., Fc gamma receptor IIB (FcγRIIB) (164, 165) and FcγRIIC (166) and confer anti-inflammatory responses against SARS-CoV-2 (Completed Clinical Trials: hyperimmune plasma NCT04321421).

### Nrf-2 Modulators vs. SARS-CoV-2 Induced Oxidative Stress

Oxidative stress is significantly induced by several viral infections inside the lungs through the downregulation of redox regulator nuclear factor-erythroid 2 related factor 2 (Nrf-2) (167). Nrf-2 is a leucine-zipper transcription factor (167) expressed predominantly in nasal epithelium, epithelial cells of lungs, and alveolar macrophages (168, 169). Disruption of Nrf-2 and Keap1 interaction triggers the activation of the anti-oxidant defense mechanism (169). For instance, Nrf-2 activation offers protection against inflammation and lung injury induced by influenza viral infections and respiratory syncytial virus (RSV) through the anti-oxidant defense pathway (170). Several viral proteins in the host cells can foster optimum levels of ROS-mediated oxidative stress to facilitate viral metabolism and the viral replication cycle without killing host cells (168, 171–174). Recent seminal studies described the active role of viruses in inhibiting the Nrf-2 pathway (175–177). For instance, the positive regulation of Nrf-2 in modulating the thiol redox system and oxidative stress for the survival of infected astrocytes was observed in *Moloney murine leukemia virus and HIV virus* (178). The HCV virus could induce the downregulation of Nrf-2 dependent NQO1, GCLC, and GPx and modulate oxidative stress (179, 180). An RSV infection mediates proteasomal degradation, deacetylation, and SUMOylation of Nrf-2 consequently causing the downregulation of NQO1, CAT, and SOD1 gene expression (181). Hence, Nrf-2 activators are potential anti-viral agents, which can be tested against the SARS-CoV-2 infection (167). Future research is highly imperative in unraveling the underlying activity of Nrf-2 for emerging SARS-CoV-2 survival by analyzing Nrf2 target genes NQO1, GCLC, and GPx. In addition, the SARS-CoV-2 mediated expression of serine and cysteine proteases in different cell lines should be investigated in relation to Nrf-2 activation, which is a beneficial strategy to combating SARS-CoV-2 pathogenesis. However, in the case of certain viral infections, it is imperative to develop Nrf-2 inhibitors to protect the host cells (182). For instance, the Marburg virus (a causative agent for lethal hemorrhagic fever) can modulate oxidative stress by activating Nrf-2 dependent signaling through the blockade of “VP-24 viral protein” binding to KEAP1 (183). Therefore, VP-24 dependent Nrf2 activation can mediate the upregulation of genes HO (heme oxygenase)-1, NQO1, and GCLM (183). In the case of Dengue virus, the viral particles could induce ER stress and activate Nrf-2 signaling, which then lead to TNF-α secretion (184). In this scenario, it is crucial to uncover any underlying mechanisms of emerging SARS-CoV-2 survival through the modulation of oxidative stress via Nrf-2 signaling in different cells of different organs including lungs (183). The prospective research studies should focus on the development of Nrf-2 modulators against SARS-CoV-2.

#### Natural Nrf-2 Modulators vs. Viral Infections

Natural products were proven to offer protection against virus-induced oxidative stress by modulating anti-oxidant defense pathways (185, 186). For instance, the administration of EGCG has mitigated viral replication of “influenza A/Bangkok/1/79 infection” by activating Nrf-2 to attenuate virus-induced oxidative stress, inflammation, and apoptosis in lung cells (186). Similarly, the cytoprotective and antioxidant efficacy of Nrf-2 was reported against PR8 influenza-A viral infection in AT-I and AT-II cells (186). Prospective research should focus on testing the efficacy of several natural products to block SARS-CoV-2 viral replication by ascertaining Nrf-2 mediated antioxidant responses. Studies have also shown the activation of host cellular transmembrane proteases (for example, serine proteases, cysteine proteases), which can further foster a prompt viral entry and viral replication in host cells by reducing Nrf-2 expression (4). Decline in proteolysis of the above proteases can actuate the propagation of several human viruses viz., Influenza, HIV, NIpah, Ebola, and Coronaviruses (SARS-CoV, MERS-CoV, SARS-CoV-2) (42, 90, 187–189). In this scenario, similar to influenza-A virus (190), it is highly important to unravel the influence of Nrf-2 expression on *TMPRSS2, and human airway trypsin-like protease* during SARS-CoV-2-mediated inflammatory conditions and oxidative stress in lungs. The downregulation of the Nrf-2 gene is correlated to serine protease activity and consequent influenza viral entry (185). Recent studies have demonstrated the efficacy of natural Nrf-2 activators viz., EGCG and sulforaphane (SFN) for blocking viral entry/viral replication as well as promoting antiviral mediators RIG-I, IFN-β, and MxA (185). In this context, it is essential to demonstrate the effects of nutritional interventions like SFN and EGCG against SARS-CoV-2 induced oxidative stress by modulating Nrf-2 signaling.

Evidence has demonstrated the use of naturally occurring Nrf2 activators for mitigating viral infections/post-viral infection induced complications. For example, α-luminol is a natural Nrf-2 activator, which confers the protection of astrocytes against the MoMuL virus (191). EGCG enhances nuclear Nrf-2 levels during Tat-induced HIV-1 infection and offers protection against virus induced oxidative stress (192). Tanshinone II A can induce upregulation of Nrf-2 expression and mitigates ROS production during Tat-induced HIV-1 infection via modulating *AMPK/Nampt/SIRT1* signaling in host cells (193). SFN enhances the phagocytic function of “HIV-infected alveolar macrophages in lungs” by activating Nrf-2 signaling, which further induces downstream antioxidant cascades (194). Lucidone is effective for the Nrf-2 mediated blockade of Dengue virus by inducing heme oxygenase-1 (195); rographolide could induce Nrf-2 induced antioxidant defenses against influenza A in lung cells (196); celastrol can mediate Nrf-2 induced antioxidant defenses against HIV-1 Tat-induced inflammation (197). Broccoli sprouts containing SFN acts as a Nrf-2 activator to reduce influenza-induced infection in lung cells (198). Bakuchiol and Rupestonic acid are phytoconstituents that confer Nrf-2 activation thereby promoting NQO1 gene expression and HO-1-mediated interferon activity to enhance antioxidant response against influenza virus in lung cells (199, 200). Curcumin is another significant compound that can modulate Nrf-2 signaling and enhance the generation of IFN-β to offer protection against the influenza virus (201). Curcumin can mitigate this viral infection by modulating *TLR2/4, p38/JNK MAPK, and NF-*κ*B pathways* (201). However, studies are required to decipher the activity of these phytochemicals against SARS-CoV-2 (Table 4). A recent report by Drăgoi (209) hypothesized that the potent natural Nrf-2 activators viz., resveratrol, SFN, curcumin, and Asea redox should be evaluated in different combinations with conventional drugs against SARS-CoV-2 infection in both *in vitro* and *in vivo* models and to further deduce a correlation between Nrf-2 activity and SARS-CoV-2 viral entry/replication.

**Table 4:** Structure and mechanism of action of naturally occurring Nrf2 modulators.

**Nrf-2 modulators effective against viruses**	**Mechanism of Action**	**Source and structure**	**References**
EGCG	Inhibits viral replication of influenza A/Bangkok/1/79 infection in lung cells Inhibits Tat-induced HIV-1 infection	A polyphenol-Dried leaves of green tea 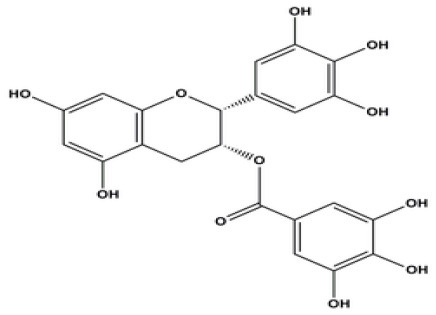	(167) (185) (202)
SFN	Inhibits viral replication by enhancing expression of Nrf-2 expression, and antiviral mediators viz., RIG-1, IFN-β, and MxA.	Isothiocyanate—cruciferous vegetables 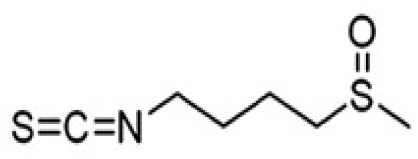	(185) (203)
α-luminol (monosodium α-luminol)	Inhibits MoMuL virus	Chemical synthesis 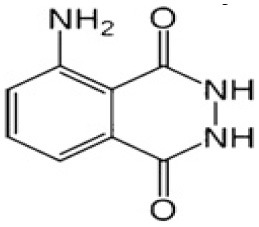	(191)
Tanshinone IIA	Inhibits Tat-induced HIV-1 via Nrf-2 upregulation	*Salvia miltiorrhiza Bunge* 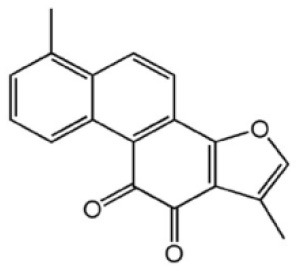	(204) (204)
Lucidone	Inhibits Dengue virus HCV (Hepatitis C Virus) growth	*Lindera erythrocarpa Makino* 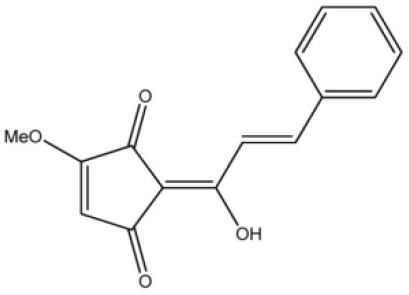	(195)
Celastrol (quinone methide triterpene)	Inhibits Tat-induced HIV-1 infection	*Tripterygium wilfordii* 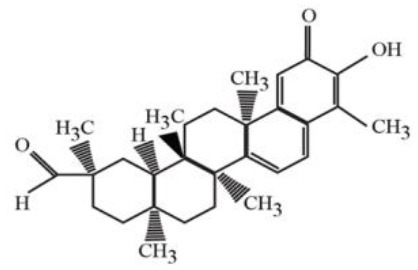	(197) (205)
Bakuchiol (phenolic isoprenoid)	Inhibits **influenza A H1N1** lung virus infection	*Psoralea corylifolia L*. 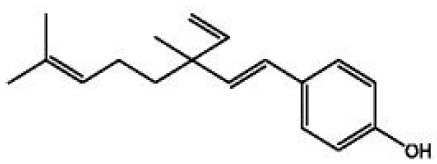	(199) (206)
Rupestonic acid (sesquiterpene)	Inhibits **influenza A (H1N1)** lung virus infection	*Artemisia rupestris L*. 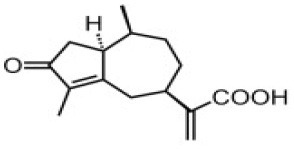	(207)
Curcumin	Inhibits **influenza A (H1N1)** lung virus infection	*Turmeric* 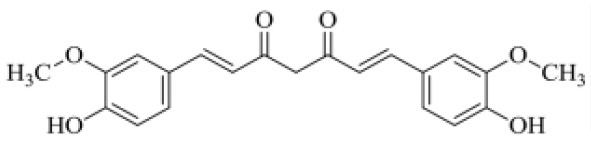	(208) (201)

#### Naturally Occurring Small Molecule Inhibitors vs. SARS-CoV-2

SARS-CoV-2 is progressively inducing a high mortality rate across the globe due to the lack of selective therapeutic interventions or vaccination. Recent reports by Lu et al. (210) and Xu et al. (148) delineated that the S-protein of SARS-CoV-2 and SARS Co-V exhibit similar 3-D pharmacophore in the receptor binding domain (RBD) of ACE-2 of human cells. COVID-19 patients are characterized by the severe viral pathogenesis due to extensive cytokine storm viz., TNF-α, IL-1β, IL-10, IFNγ, and MCP-1 in infected lung tissues (48). A report by Chen and Du (211) hypothesized that the phyto-constituents such as “*baicalin, scutellarin, hesperetin, nicotianamine, and glycyrrhizin*” may deliver anti-SARS-CoV-2 effects. Hesperetin glycoside abundant in citrus fruits, which can inhibit the SARS-CoV 3CLpro (212). The activity of this molecule must be examined against serine/cysteine proteases, which support SARS-CoV-2 viral entry/replication. Traditional citrus flavonoids were reported to have a potential to act against SARS-CoV-2 as studied by molecular docking studies. Molecular docking simulations, LC-MS studies described the efficacy of citrus flavonoids (ex. naringenin) in binding to ACE-2, and mitigating inflammation-induced lung injury by the SARS-CoV-2 virus (213). Further studies should evaluate these compounds in preclinical models to determine the safety and efficacy against the SARS-CoV-2 infection.

Natural products such as di/tri-terpenoids, lignoids were proven to inhibit the viral replication of coronaviruses *in vitro* (214); griffithsin could block coronaviral entry by binding to the SARS-CoV spike glycoprotein (215). TSL-1 can block coronaviral entry/replication; Leaf extracts of *Toona sinesis Roem* effectively blocked SARS-CoV replication (57). Betulinic acid, savinin can act as competitive inhibitors against SARS-CoV 3CL protease to block viral entry (214). The research gap must be filled to develop nutritional therapeutic interventions by investigating the efficacy of these phytochemicals against SARS-CoV-2 viral entry. Seeds of *Psorelia corylifolia* exhibit inhibitory effects against the SARS-CoV papain-like protease required for coronavirus entry/replication. The efficacy of these molecules should be examined against SARS-CoV-2 (216) (Table 5). The active site pockets of main proteases such as 6LU7 and 2 GTB in SARS-CoV-2 are reported to be involved in conferring viral entry/fusion; hence, these sites should be considered as the potential drug targets against SARS-CoV-2 (66). A molecular docking study by Khaerunnisa et al. (65) reported the predicted efficacy of bioactive compounds against above SARS-CoV-2 main protease (Mpro) sites viz., “*nelfinavir, lopinavir, kaempferol, quercetin, luteolin-7-glucoside, demethoxycurcumin, naringenin, apigenin-7-glucoside, oleuropein, curcumin, catechin, epicatechin-gallate, zingerol, gingerol, and allicin”* (Table 5).

**Table 5:** Structure and mechanism of action of NSMIs identified against SARS-CoV2 using molecular docking studies.

**NSMIs Identified using Molecular Docking**	**Mechanism of Action**	**Source and Structure**	**References**
Baicalin (a flavonoid)	Predicted to exhibit a capacity for binding to ACE-2 for inducing anti-SARS-CoV-2 effects	*Scutellaria baicalensis* 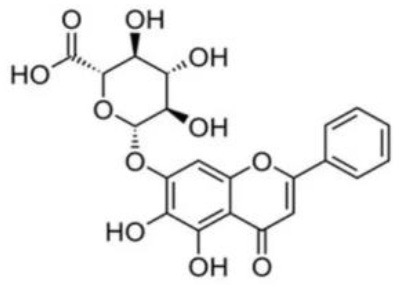	(211, 217)
Scutellarin (a flavone glycoside)	Predicted to exhibit a capacity for binding to ACE-2 to induce anti-SARS-CoV-2 effects	*Erigeron breviscapus* 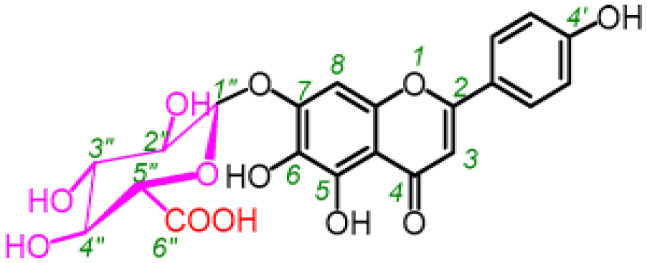	(211, 218)
Nicotianamine	Predicted to exhibit a capacity for binding to ACE-2 to induce anti-SARS-CoV-2 effects	Leaves of *L. chinense* *Fagus sylvatica, Avena sativa* *Oryza sativa, Soybean* 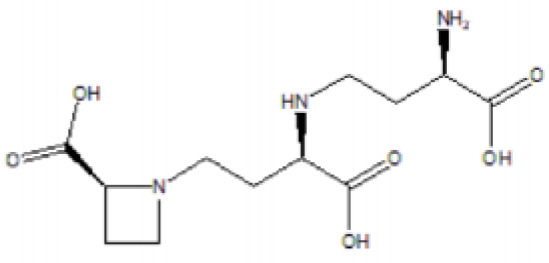	(211, 219, 220)
Glycyrrhizin	Predicted to exhibit a capacity for binding to ACE-2 to induce anti-SARS-CoV-2 effects Note: Yet to be examined against SARS-CoV-2 infection	*Liquorice root (Glycyrrhiza radix)*, 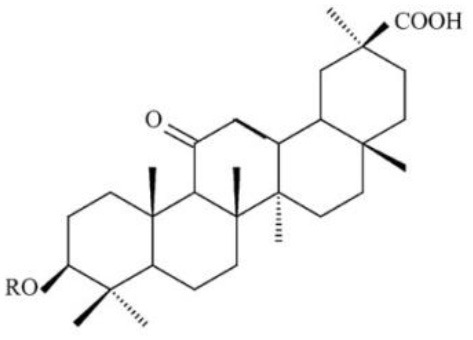	(211)
Hesperetin glycoside	Potent inhibitor of SARS-CoV 3CLpro Note: Yet to be examined against SARS-CoV-2 infection	*Citrus aurantium* *Citri Reticulatae Pericarpium* 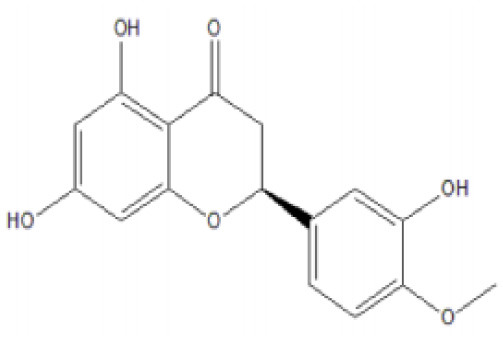	(211, 212)
Naringenin	Binds to ACE-2, a receptor for SARS-CoV-2	*Citrus wilsonii Tanaka* 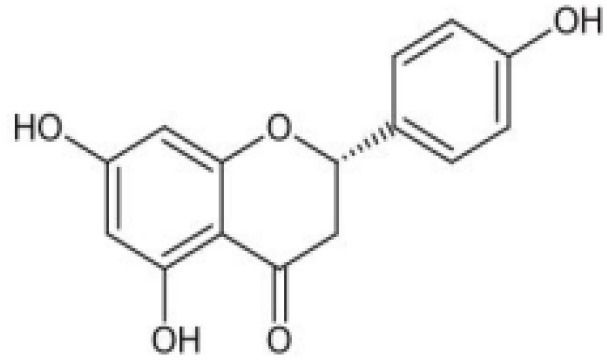	(211, 213)
Betulinic acid	Competitively inhibits SARS-CoV 3CL protease	*Outer bark of the birches (Betula)* 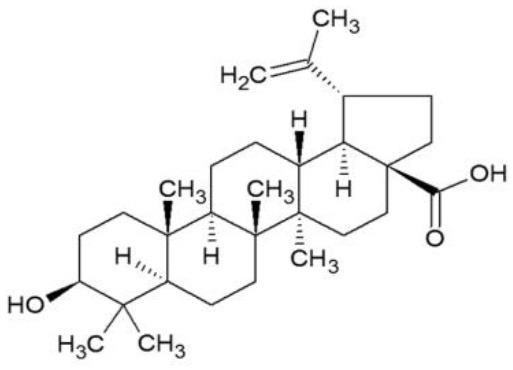	(214, 221)
Griffithsin	Binds to the SARS-CoV spike (S) -protein and inhibit viral entry	*Red algae (Griffithsia species)* 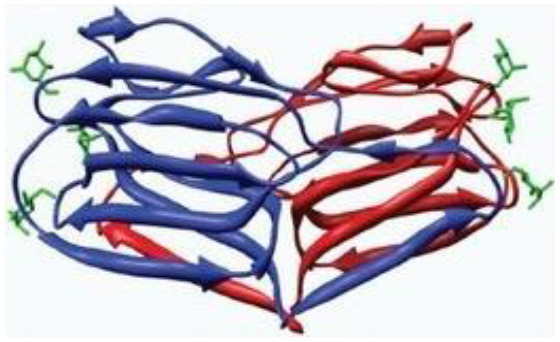	(215, 222)
Savinin	Competitively inhibits SARS-CoV 3CL protease	A Lignan from *Pterocarpus santalinus* 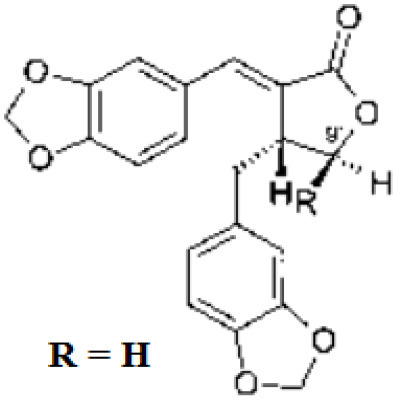	(214)
Quercetin	Predicted to inhibit SARS-CoV-2 6LU7 Main protease (Mpro)	*Red grapes, citrus fruit* 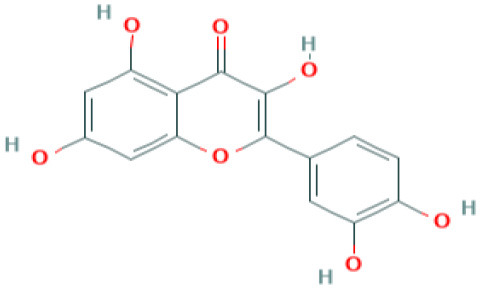 *(Pubchem)*	(65)
Kaempferol	Predicted to inhibit SARS-CoV-2 6LU7 Main protease (Mpro)	*Delphinium* 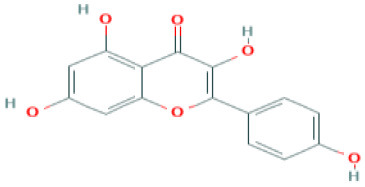 *(Pubchem)*	(65)
Allicin	Predicted to inhibit SARS-CoV-2 6LU7 Main protease (Mpro)	Garlic *(Allium sativum)* 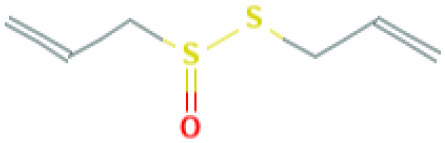 *(Pubchem)*	(65)
Gingerol	Predicted to inhibit SARS-CoV-2 6LU7 Main protease (Mpro)	Ginger *(Pubchem)* 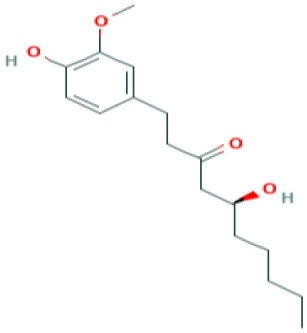	(65)
Catechin	Predicted to inhibit SARS-CoV-2 6LU7 Main protease (Mpro)	Green tea 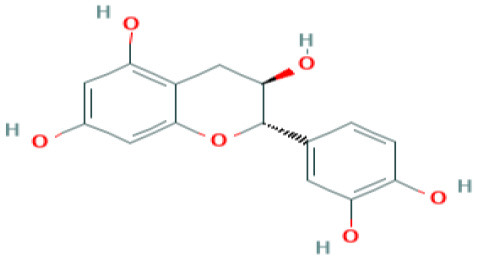 *(Pubchem)*	(65)
Epicatechingallate	Predicted to inhibit SARS-CoV-2 6LU7 Main protease (Mpro)	*Rhubarb, Parapiptadenia rigida* 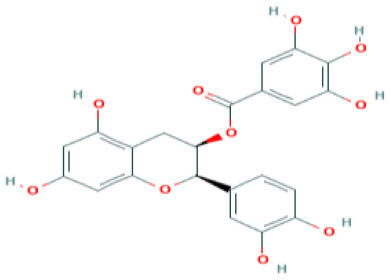 *(Pubchem)*	(65)
Curcumin	Predicted to inhibit SARS-CoV-2 6LU7 Main protease (Mpro)	*Curcumin longa* 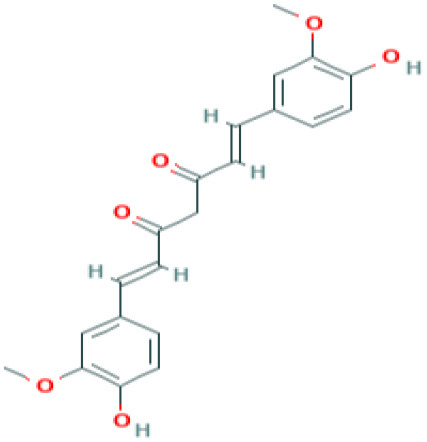 *(Pubchem)*	(65)
Apigenin-7- glucoside	Predicted to inhibit SARS-CoV-2 6LU7 Main protease (Mpro)	*Parsley* 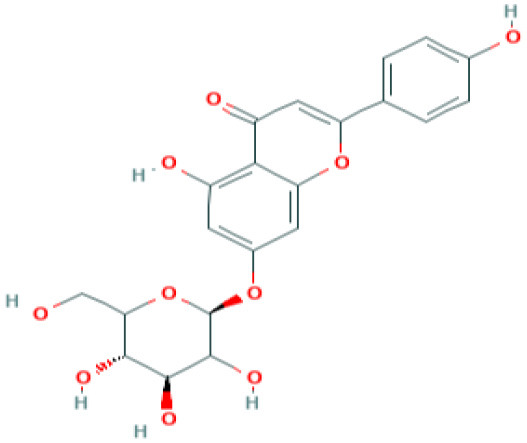 *(Pubchem)*	(65)
Luteolin-7- glucoside	Predicted to inhibit SARS-CoV-2 6LU7 Main protease (Mpro)	Leaves of *Capsicum annuum* 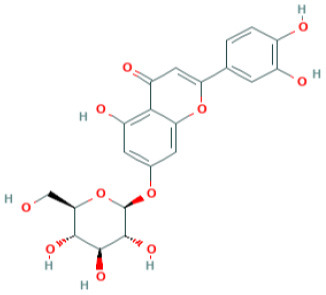 *(Pubchem)*	(65)

## Conclusions

The life-threatening consequences of the COVID-19 pandemic remain high due to lack of selective targeted therapies and vaccination strategies. This is primarily due to extreme genomic variability of RNA viruses as well as variations in the host-cell invading mechanisms. Hence, this review benefits virologists, medical scientists, and cell biologists to ascertain and develop NSMIs, Nrf-2 modulators, and clinically viable vaccines to combat this devastating SARS-CoV-2 strain. However, many more preclinical and clinical studies are required to uncover the therapeutic efficacy of potential phytochemicals, natural Nrf2 modulators, and several NSMIs against the SARS-CoV-2 infection. Furthermore, studies are also warranted to overcome ADE responses, and Th-2 immunopathology for the development of safe and efficacious vaccines against SARS-CoV-2. In summary, this review provides an overview on the existing knowledge and shows directions to various areas that require immediate attention.

## Author Contributions

NB, SS, and SM: idea development, data collection, manuscript preparation, writing, and proof reading. AS, VN, and LM: cross referencing, data collection, and proof reading. SM, GA, and RR: editing, literature search, and proof reading. SS: execution of the literature search. All authors contributed to the article and approved the submitted version.

## Conflict of Interest

GA is employed by GALLY International Biomedical Research & Consulting LLC. The remaining authors declare that the research was conducted in the absence of any commercial or financial relationships that could be construed as a potential conflict of interest.

## Abbreviations

RTIs: Respiratory tract infections
ORF: Open reading frame
TMPRSS2: Transmembrane serine protease
ADAM17: Disintegrin and metallopeptinase-containing domain 17
SARS-CoV: Severe respiratory syndrome-coronavirus
SARS-CoV-2: Severe respiratory syndrome-Coronavirus-2 (COVID-19)
GM-CSF: Granulocyte–macrophage colony-stimulating factor.
